# Drug prescription goals in primary care: a cross-sectional study

**DOI:** 10.1186/s12913-019-4870-y

**Published:** 2020-01-02

**Authors:** Louis Bernard, René Ecochard, François Gueyffier, Laurent Letrilliart

**Affiliations:** 1 Univ. Lyon, Université Claude Bernard Lyon 1, Collège universitaire de médecine générale, F-69008 Lyon, France; 2 Hospices Civils de Lyon, Service de Biostatistique-Bioinformatique, Lyon, France; 3 Université Lyon 1, Villeurbanne, France; 4 Laboratoire de Biométrie et Biologie Évolutive, Équipe Biostatistique-Santé, CNRS, Villeurbanne, France; 5 UMR5558, service de pharmacologie et toxicologie, faculté Laennec, Lyon, France; 6 Univ. Lyon, Université Claude Bernard Lyon 1, Collège universitaire de médecine générale, F-69008 Lyon, France; 7 Univ. Lyon, Université Claude Bernard Lyon 1, HESPER EA 7425, F-69008 Lyon, France

**Keywords:** Goals, Drug prescription, General practice, Primary care, Observational study

## Abstract

**Background:**

Care goals are often implicit, although their identification is a key element of any prescription process. This study aimed to describe the clinical goals of drug prescriptions in general practice, their determinants and the agreement between physicians and patients.

**Methods:**

This was a cross-sectional study conducted by 11 resident trainees acting as observers in 23 general practices. The residents recorded the indication and main physician’s goal for all drugs prescribed during five consultation days in each practice in December 2015, and the main patient’s goal for a sub-sample of consultations. We used an eight-category generic classification of prescription goals, including three specific (mortality, morbidity and cure), three non-specific (symptoms, quality of life, functioning) and two non-specified (other goal, no goal) categories. Analyses were based on a multivariable, multilevel model and on the kappa statistic applied to the sub-sample of consultations.

**Results:**

The sample encompassed 2141 consultations and 5036 drugs. The main physicians’ goal of drug prescriptions was to relieve symptoms (43.3%). The other goals were to decrease the risk of morbidity (22.4%), to cure disease (11.7%), to improve quality of life (10.6%), to decrease the risk of mortality (8.5%) and to improve functioning (1.8%). The choice of a specific goal was more frequent in patients with the following characteristics: over 50 (OR [1.09;1.15]), of male gender (OR [1.09;1.39]), with full financial coverage for a long-term condition (OR [1.47;1.97]), known by the physician (OR [1.19;2.23]), or with a somatic health problem (OR [2.56;4.17]). Cohen’s kappa for drug prescription goals between the patients and the physicians was 0.26 (0.23–0.30).

**Conclusions:**

Physicians’ goals are poorly shared with patients. It remains to be assessed whether it is possible to collect and discuss information on prescription goals on a daily basis.

## Background

The medical process usually starts with the identification of a health problem and the determination of a diagnosis. It is only after those steps, and according to the diagnosis that has been established, that a therapeutic decision is made. This decision, which aims to have a positive effect on the health of the patient, requires setting one or several care goals that can be somehow formalized. The identification of these goals is acknowledged to be one of the key elements of any prescription process [1]. Explaining these goals can help the practitioner make sure they are clinically relevant and can help the patient accept and stick to the treatment [2, 3]. Discussing the care goals contributes to a shared medical decision [4, 5] in accordance with patients’ needs, preferences and values [6–8]. It enables the patient to prioritize his or her expectations [9] and the practitioner to have the care goals coincide with the most important expectations of the patient [10, 11]. Indeed, the therapeutic proposals should meet patients’ preferences [12, 13].

Within the framework of evidence-based medicine, the therapeutic decision should be made according to studies based on the highest level of evidence available. The studies can lead to different conclusions depending on the outcome criteria that are considered. Making a therapeutic decision based on the results of a study implies that the therapeutic goal determined for the patient coincides with the outcome of the study. Sharing a therapeutic goal or a set of goals with the patient is a way for the physician to quantify the therapeutic benefit and therefore personalize it [14, 15]. A clinical approach that includes explicit goals, measurable at the patient level, allows for the possibility to use them for monitoring treatment and for assessing care quality in terms of results [8, 9, 16, 17].

Two primary care studies have shown that less than half of the patients with chronic conditions had already discussed the goals of their treatments with their doctors [18, 19]. However, these studies did not mention the type of care goals they took into account, and in particular whether the goals were intermediary—in order to improve biological or physiological markers—or clinical [4, 20]. A couple of studies have described the care goals of patients and/or practitioners, but in selected groups of multimorbid or end-of-life patients, and these studies did not link the care goals to a specific health problem or treatment [10, 21–24].

Our primary objective was to describe the physicians’ clinical goals of primary care drug prescriptions according to the patients’ characteristics and the prescribed drugs. Our secondary objectives were to measure the level of agreement between the patients’ and the physicians’ goals and to determine the factors influencing physicians’ goals.

## Methods

The study was a multicenter, cross-sectional regional study conducted in French general practice. It included 23 centers using the practices of university trainers associated with the College of General Practice of the University of Lyon 1.

### Inclusion criteria

The data were collected by 11 general practice residents under the supervision of university trainers, who acted as passive observers on the days of data collection. Each resident trainee investigated 2 or 3 different practices. The inclusion days represented approximately one training day by trainer per week. All the patients encountered at the office over a period of 5 days in each practice from November 2, 2015 to January 6, 2016 were briefly informed at the start of the encounter on the study aim and protocol and included after providing their consent. The eight patients who refused to participate or to be examined in the presence of the resident were not included.

### Data collection

The resident investigators had training sessions on how to collect and enter the data. They were given a checklist with the various procedures necessary for data collection and entry.

The data from the consultations was collected as free text on a paper questionnaire document (See Additional file 1). It focused on the following variables: patient age, gender, socio-professional class and medical fee exemption status (full financial coverage by the national public health care insurance for long-term conditions or for low income), new or returning patient, health problems managed by the physician, and for each health problem assessed, the prescribed drugs—first prescription or renewed prescription. After each consultation, once the patient left, the investigating resident asked the physician to state the main goal of each of the prescribed drugs according to a generic list. This classification included the following eight predefined goal categories: to decrease the risk of mortality (all-cause or cause-specific); to decrease the risk of morbidity (disease or complications); to cure or provide remission of disease; to relieve symptoms; to improve or maintain quality of life; to improve or maintain functioning; other goal; no goal.

The following data were collected on the university trainers: age, gender, work environment (rural, semi-rural, urban), and type of practice (solo, group practice, multidisciplinary group/health care center).

During 1 day per practice, each resident investigator asked the patient, in addition to the physician, to explain the main goal of his prescription according to the same list of categories (with a wording adapted to patients’ level of understanding). These data were collected after each consultation through a short interview performed out of the consultation room (and then without the prescribing physician). This classification had been developed empirically by a group of French and Belgium physicians, members of the Wonca International Classification Committee, aiming at producing a simple set of discrete, exhaustive and mutually exclusive categories covering primary care goals [25].

### Data entry

The resident investigators entered the data they had collected on the paper questionnaire into a central database that was accessible via a dedicated website. The data about the health problems managed were entered after being coded according to the French version of the International Classification of Primary Care (ICPC-2) [26], with the help of an online coding software [27]. With the support of a search engine, the drugs that had been prescribed were entered into the Thériaque database [28], which includes the Anatomical Therapeutic Chemical (ATC) classification of drugs [29]. A subsample of the consultations was subject to a double entry to verify the accuracy of the entry of drugs and goals.

### Data analyses

Data analyses consisted of describing the frequency of the various drug prescription goals and their distribution according to the characteristics of the physicians, the patients and the health problems managed. The ICPC-2 classification is organized with 17 chapters, including 15 chapters based on body systems for somatic health problems, one chapter for psychological problems (P) and one chapter for social problems (X) [26]. Long-term (i.e. chronic) conditions were identified using a subclassification of ICPC-2 [30].

Physicians’ prescription goals were compared according to patients’ characteristics using the chi-square test. Drugs were analyzed according to the 4th level of the ATC classification. The influence of the factors dependent on the physician, the patient and every health problem managed was estimated with univariate analyses and then with a multivariable hierarchical logistic regression model, using the R software [31]. This model was built by arbitrarily grouping the eight categories of the classification of prescription goals into a dichotomous variable that distinguished specific goals (mortality, morbidity and cure) and non-specific goals (symptoms, quality of life, functioning), after excluding the “other goals” and “no goal” categories. This variable was used as the explained variable of the model. The level of agreement between patients’ and their physicians’ goals was estimated with the Cohen’s kappa test.

## Results

Our sample encompassed 2141 consultations, 3319 health problems managed, and 5036 drugs prescribed (i.e. 2.36 drugs per consultation [95% confidence interval: 2.26–2.47]), along with their corresponding goals. Eight patients were not included at their request. The characteristics of physicians and patients are presented in Table 1.

**Table 1:** Patients’ and physicians’ characteristics

	*n* (%)	
Patients		
Age		
≤ 14	372	(17.4)
15–44	616	(28.8)
45–74	840	(39.2)
75–97	313	(14.6)
Gender		
Males	923	(43.1)
Females	1218	(56.9)
Patient known by physician		
Yes	2017	(94.2)
No	124	(5.8)
Medical fee exemption status		
For long-term condition	410	(19.1)
For low income	96	(4.5)
Physicians		
Gender		
Males	16	(69.6)
Females	7	(30.4)
Age (yrs)		
31–39	5	(21.7)
40–49	5	(21.7)
50–59	9	(39.2)
59–66	4	(17.4)
Work environment		
Rural	9	(39.0)
Semi-rural	8	(34.8)
Urban	6	(26.2)
Type of practice		
Multidisciplinary group/health care center	6	(26.0)
Group	14	(60.9)
Solo	3	(13.1)

### Distribution of prescription goals

The main goal of drug prescription by the physicians was to relieve symptoms (43.3%) before decreasing the risk of morbidity (22.4%), curing or providing remission of disease (11.7%), improving quality of life (10.6%), decreasing the risk of mortality (8.5%) and improving functioning (1.8%). Some prescription goals were classified in the “other goal” category (1.2%), especially for the prescription of contraception. Exceptionally, the physician reported no goal (0.5%), which could happen for granting a drug request for the patient’s family medicine cabinet.

Physicians’ goals for prescribing drugs were associated with patients’ gender and age, physicians’ knowledge of the patient and the existence of full financial coverage for a long-term condition. In particular, for male patients over 45 who were already known by the physician or had full financial coverage for a long-term condition, the goals to decrease mortality and morbidity were more frequent, whereas the goals to relieve symptoms were less frequent (Table 2).

**Table 2:** Distribution of physicians’ prescription goals according to patients’ characteristics

	Mortality		Morbidity		Cure		Symptom		Quality of life		Functioning		Other or none		Total	
Total	429	(8.5%)	1128	(22.4%)	587	(11.7%)	2183	(43.3%)	534	(10.6%)	91	(1.8%)	84	(1.7%)	5036 (100%)	
Gender																*p* < 0.01
Male	213	(10.0%)	564	(26.4%)	213	(10.0%)	858	(40.2%)	217	(10.2%)	41	(1.9%)	28	(1.3%)	2134 (100%)	
Female	216	(7.4%)	564	(19.4%)	374	(12.9%)	1325	(45.7%)	317	(10.9%)	50	(1.7%)	56	(1.9%)	2902 (100%)	
Age																*p* < 0.01
0–44	44	(2.7%)	151	(9.1%)	271	(16.4%)	970	(58.7%)	151	(9.1%)	24	(1.5%)	42	(2.5%)	1653(100%)	
≥45	385	(14.7%)	977	(31.6%)	316	(6.7%)	1213	(31.9%)	383	(11.7%)	67	(2.1%)	42	(1.3%)	3383 (100%)	
Patient known by physician																*p* < 0.01
Yes	420	(8.7%)	1103	(23.0%)	561	(11.7%)	2046	(42.6%)	512	(10.7%)	83	(1.7%)	79	(1.6%)	4804 (100%)	
No	9	(3.9%)	25	(10.8%)	26	(11.2%)	137	(59.1%)	22	(9.5%)	8	(3.4%)	5	(2.2%)	232 (100%)	
Long term condition																*p* < 0.01
Yes	285	(16.6%)	564	(32.9%)	112	(6.5%)	492	(28.7%)	200	(11.7%)	39	(2.3%)	21	(1.2%)	1713 (100%)	
No	144	(4.3%)	564	(17.0%)	475	(14.3%)	1691	(50.9%)	334	(10.1%)	52	(1.6%)	63	(1.9%)	3323 (100%)	
Low income																NA
Yes	18	(8.2%)	29	(13.2%)	25	(11.4%)	108	(49.1%)	36	(16.4%)	0	(0%)	4	(1.8%)	220 (100%)	
No	411	(8.5%)	1099	(22.8%)	562	(11.7%)	2075	(43.1%)	498	(10.3%)	91	(1.7%)	91	(1.7%)	4816 (100%)	

NA Chi-square test not available due to a theoretical sample < 5

The drugs most often prescribed with the goal of decreasing mortality and morbidity were cardiovascular system drugs (ATC class C). The drugs most often prescribed with the goal of curing disease or providing remission were penicillins with extended spectrum (Table 3). As far as symptoms and quality of life improvements were concerned, the anilides class (including paracetamol) prevailed.

**Table 3:** The most frequent 4th level ATC sub-classes according to prescription goals

Goal	ATC sub-class	*n* (%)
Mortality (*n* = 429)	Platelet aggregation inhibitors excl. Heparin (B01AC)	51 (11.9%)
	HMG CoA reductase inhibitors (statin) (C10AA)	48 (11.2%)
	Beta blocking agents, selective (C07AB)	32 (7.5%)
	ACE inhibitors, plain (C09AA)	30 (7.0%)
	Angiotensin II antagonists, plain (C09CA)	22 (5.1%)
Morbidity (*n* = 1128)	HMG CoA reductase inhibitors (statin) (C10AA)	106 (9.4%)
	Vitamin D and analogues (A11CC)	74 (6.6%)
	ACE inhibitors, plain (C09AA)	65 (5.8%)
	Beta blocking agents, selective (C07AB)	59 (5.2%)
	Calcium channel blockers, dihydropyridine derivatives (C08CA)	54 (4.8%)
	Platelet aggregation inhibitors excl. Heparin (B01AC)	54 (4.8%)
Cure (*n* = 587)	Penicillins with extended spectrum (J01CA)	59 (10.1%)
	Glucocorticoids (H02AB)	38 (6.5%)
	NSAID propionic acid derivatives (M01AE)	31 (5.3%)
	Selective serotonin reuptake inhibitors (N06AB)	29 (4.9%)
	Imidazole and triazole derivatives for topical use (D01AC)	25 (4.3%)
Symptom (*n* = 2183)	Anilides^a^ (N02BE)	559 (25.6%)
	NSAID propionic acid derivatives (M01AE)	121 (5.5%)
	Proton pump inhibitors (A02BC)	113 (5.2%)
	Corticosteroids for nasal use (R01AD)	105 (4.8%)
	Other drugs for functional gastrointestinal disorders (A03AX)	78 (3.6%)
Quality of life (*n* = 534)	Anilides^a^ (N02BE)	51 (9.6%)
	Benzodiazepine derivatives (N05BA)	32 (6.0%)
	Benzodiazepine related drugs (N05CF)	30 (5.6%)
	Selective serotonin reuptake inhibitors (N06AB)	30 (5.6%)
	Proton pump inhibitors (A02BC)	25 (4.7%)
Functioning (*n* = 91)	Other anti-inflammatory and antirheumatic agents, non-steroids (M01AX)	7 (7.7%)
	Vitamin D and analogues (A11CC)	4 (4.4%)
	Anilides^a^ (N02BE)	4 (4.4%)
	Other nasal preparations (R01AX)	4 (4.4%)
Other (*n* = 60)	Progestogens and estrogens, fixed combinations (G03AA)	9 (15.0%)
	Progestogens and estrogens, sequential preparations (G03AB)	6 (10.0%)
	Bacterial and viral vaccines, combined (J07CA)	3 (5.0%)
None (*n* = 24)	Vitamin D and analogues (A11CC)	4 (16.7%)
	Antiseptics biguanides and amidines (D08AC)	3 (12.5%)
	Natural opium alkaloids (N02AA)	2 (8.3%)
	Anilides^a^ (N02BE)	2 (8.3%)
	Mucolytics (R05CB)	2 (8.3%)

^a^Including paracetamol

**Fig. 1:**
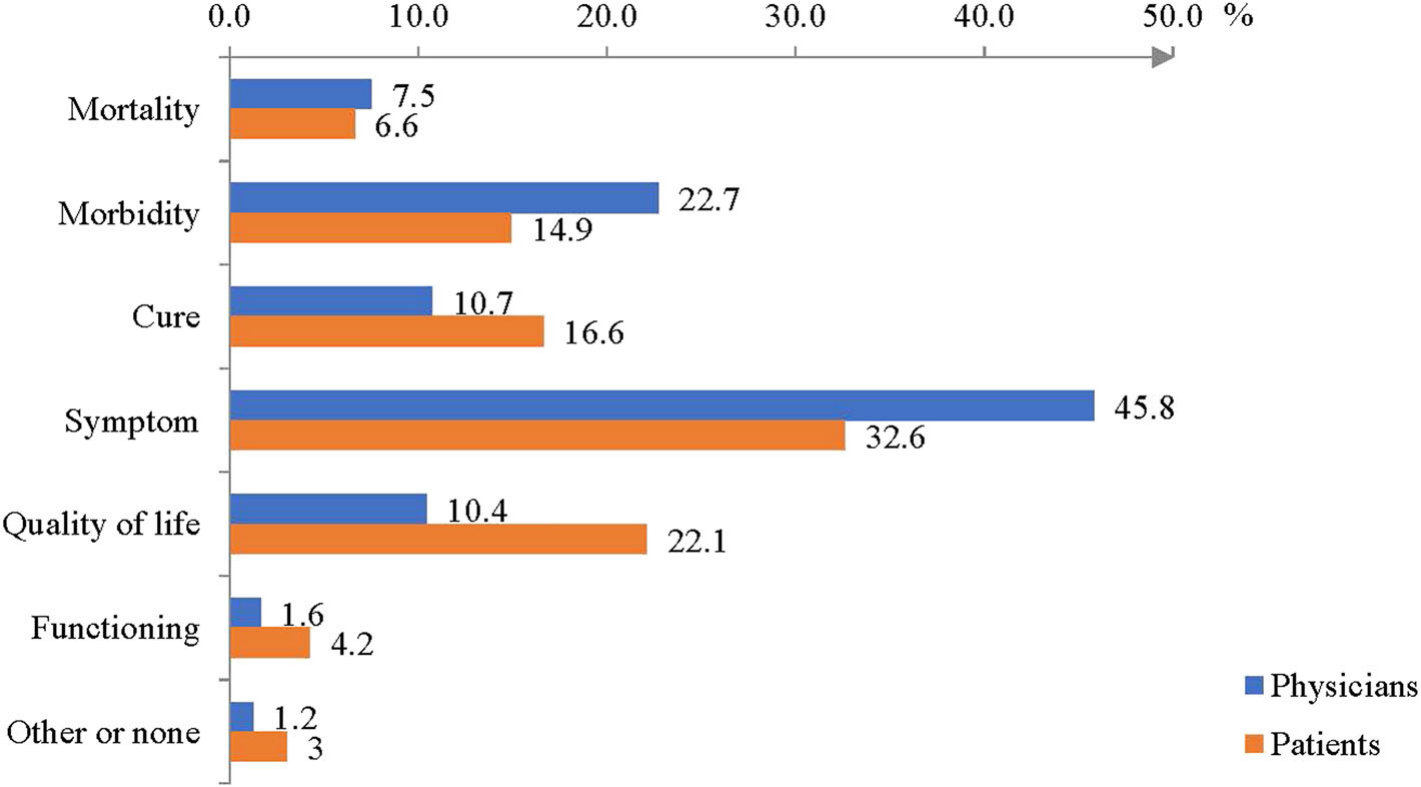
Distribution of patients’ and physicians’ prescription goals

Patients’ and physicians’ goals were compared from a sub-sample of 355 consultations (16.6%) and 1129 drugs and goals (22.4%). They reported the same goal in 488 prescriptions (44.2%). The Cohen’s kappa coefficient between patients’ and physicians’ goals was equal to 0.26 (0.23–0.30), corresponding to a poor level of agreement. The difference in assessment was highest for the goal of improving quality of life (22.1% the patients vs. 10.4% for physicians) (Fig. 1).

### Reliability of the data entry

A sub-sample of 190 consultations (8.9%) was entered twice. Among the 606 prescribed drugs, 18 were different between the two entries (3.0%)—14 because of a different drug and 4 because of a missing drug. Among the 606 prescription goals, 19 were different between the two entries (3.1%)-15 because of a different goal and 4 because of a missing goal.

### Determinants of physicians’ prescription goals

Once the goals were grouped in two main categories—specific and non-specific goals—the multivariate analyses showed that drugs were prescribed more frequently with a specific goal when the patient was over 50 (OR = 1.12), of male gender (OR = 1.23), had full financial coverage for a long-term condition (OR = 1.70), was already known by the physician (OR = 1.63) or had a somatic health problem managed (OR = 3.23) (Table 4).

**Table 4:** Determinants of physician’s choice of a specific goal

	Specific goals (*n* = 2144)		Non-specific goals (*n* = 2808)		Univariate analysis		Multivariable analysis^a^	
					OR	[IC 95%]	OR	[IC95%]
Characteristics of the health problem managed								
Psycho-social	93	(4.3%)	323	(11.5%)	1.00		1,00	
Somatic	2051	(95.7%)	2485	(88.5%)	2.86	[2.27; 3.57]	3.23	[2.56; 4.17]
Characteristics of the patient								
Age (yrs)								
< 50	554	(25.8%)	1285	(45.8%)	1.00		1.00	
≥ 50	1590	(74.2%)	1523	(54.2%)	2.42	[2.14; 2.73]	1.12	[1.09; 1.15]
Gender								
Females	1154	(53.8%)	1692	(60.3%)	1.00		1,00	
Males	990	(46.2%)	1116	(39.7%)	1.30	[1.16; 1.45]	1.23	[1.09; 1.39]
Long-term condition								
No	1183	(55.2%)	2077	(74.0%)	1.00		1,00	
Yes	961	(44.8%)	731	(26.0%)	2.31	[2.05; 2.60]	1.70	[1.47; 1.97]
Low income								
Yes	72	(3.4%)	144	(5.0%)	1.00		1.00	
No	2072	(96.6%)	2664	(95.0%)	1.56	[1.17; 2.08]	1.30	[0.95; 1.79]
Patient known by the physician								
No	60	(2.8%)	167	(5.9%)	1.00		1.00	
Yes	2084	(97.2%)	2641	(94.1%)	2.20	[1.63; 2.97]	1.63	[1.19; 2.23]
Characteristics of the physician								
Work environment								
Urban	539	(25.1%)	789	(28.1%)	1.00		1.00	
Rural or semi-rural	1605	(74.9%)	2019	(71.9%)	1.16	[1.02; 1.32]	1.20	[0.98; 1.47]

^a^Adjusted to physician’s age and center

## Discussion

The physicians’ main goal of prescribing drugs was mostly to relieve symptoms (43.3%). The second goal was to decrease the risk of morbidity (22.4%), and the other goals were to cure disease (11.7%), to improve quality of life (10.6%), to decrease the risk of mortality (8.5%) and to improve functioning (1.8%). These goals varied according to patient’s gender and age, physicians’ knowledge of the patient, and a full financial coverage for a long-term condition. After grouping the goals, the multivariate analyses showed that physicians’ choice of a specific goal was more frequent in male patients, patients over 50, patients with full financial coverage for a long-term condition, patients who were known by the physician and patients who had a somatic health problem. Patients’ and physicians’ agreement on drug prescription goals was poor.

To the best of our knowledge, this study is the first attempt to describe the clinical goals of drug prescription in an unselected sample of patients attending general practice. It shows the diversity of the physician’ goals beyond the predominance of symptom relief, and the influence of various characteristics of the patient. In addition, the low agreement level between physicians’ and patients’ goals highlights the likely limited extent of shared decision on drug prescription in general practice and questions patient-centeredness of the care provided. Health care currently faces a paradigm shift from a problem-oriented model to a goal-oriented approach [32]. In this context, the discussion and sharing of drug prescription goals represents a challenge for both physicians and patients at a time when even specifying the indications of the drugs prescribed remains to be achieved by physicians [33].

### A prevailing symptomatic goal

Almost half of drugs (43.3%) are prescribed to relieve a symptom. A symptom can fit into a syndrome or an illness or can be isolated. When the symptom is related to a well-identified illness, the physician can be led to prescribe several drugs with different goals. For instance, in the case of a bacterial throat infection, antibiotics can be prescribed to reduce the risk of complications, and a painkiller can be prescribed to relieve symptoms [34]. In contrast, the health problem can correspond to an isolated symptom, when a more accurate diagnosis cannot be made during the encounter, which is the case for at least 20% of health problems assessed [35, 36]. If an isolated symptom persists in time without being related to a specific illness, it becomes a medically unexplained symptom [37]. In these two situations, a symptomatic treatment is essential [38] in a patient-centered perspective [39]. Since the absence of an accurate etiological diagnosis is a cause for patient dissatisfaction [40], the physician is confronted to a double challenge: to satisfy patients’ need to get an interpretation of their symptoms and to avoid unjustified diagnostic escalation [41].

In almost a third of the cases, the drugs prescribed to relieve symptoms were either paracetamol or non-steroidal anti-inflammatory drugs, which had probably been prescribed with an analgesic purpose. In France, most of these drugs do not require a medical prescription, and pharmacists are involved in their delivery over the counter [42]. There is probably an opportunity for a larger proportion of the mild and isolated symptoms to be addressed by pharmacists and nurses at an early stage in the healthcare pathway, leaving more time for general practitioners to manage more complex cases [43].

As far as returning patients over 45 and with a long-term condition were concerned, physicians’ goals to decrease mortality and morbidity were more frequent, whereas the goal to relieve symptoms was less frequent. Most of the time, these three categories correspond to the treatment of chronic conditions for which morbidity and mortality are high. The goals of decreasing mortality and morbidity are more frequent with male patients than with female patients, as opposed to the goal of relieving symptoms. This finding may be because men more frequently suffer from cardiovascular diseases [44], for which drug treatments have proven efficient in decreasing morbi-mortality [45, 46]. The influence of physician’s knowledge of the patient toward a more specific drug prescription goal may be due to the better delineation of the health problem diagnosis, allowing for a more specific treatment.

We observed that drug prescriptions for psycho-social problems managed in general practice were more frequently associated with non-specific goals than for somatic problems. These complex problems are dominated by depression, before anxiety and sleep disturbance [35, 36], which possibly require drugs, psychological and lifestyle interventions. As depression is frequently situational (i.e. reactive to stressful social circumstances), general practitioners often consider that the prescription by itself has a symptomatic effect but is not enough to cure the patient [47]. In addition, there is no real consensus on the concept of remission or recovery from depression [48], and it is recommended not to assess antidepressant effectiveness with categorical outcomes such as remission rates [49].

### A low level of shared decision making

The low level of agreement observed in our study between physicians’ and patients’ goals (Cohen’s kappa: 0.26) suggests that shared decision making is not yet fully effective in general practice. A study conducted with frail elderly patients has already shown that the agreement between the clinician and the caregiver about the most important goal in a list of six care goals (day-to-day functioning, safety, emotional and behavioral issues, medical issues, social support, and caregiver stress) was low (Cohen’s kappa: 0.20) [22]. Prescription goals are probably still quite implicit in the medical process [9]. A Canadian survey showed that less than half of the patients with a chronic condition have already discussed the goals of their treatment with their physician [18]. Although shared decision-making is regarded as an ethical obligation [50, 51], various barriers have been identified [7]. One of them is the difficulty for the patients to understand technical data. For this reason, some authors promote the idea of re-engineering the shared decision making by asking the patients to prioritize their care goals. In practice, rather than asking patients to choose a specific treatment or test, the physician should ask them to prioritize their goals, so that he can turn them into care processes [7]. One limitation of this approach is the lack of a validated care goals data collection system. Unfortunately, the outcome criteria used in drug trials usually do not meet the patients’ preferences, which can generate discrepancies between the scientific data available and patients’ care goals.

### An operational classification

It is common to make a distinction between treatments with preventive, curative, symptomatic and palliative aims [4]. Such classification remains too simple if we consider that the goal of prevention can encompass the prevention of mortality and morbidity and that the palliative goal can encompass the goal of relieving patients’ symptoms and improving their quality of life or functioning. Instead of those traditional goals, DL Sackett et al. have suggested using more concrete and precise goals representing the ultimate objectives of treatment, namely, to cure, prevent a recurrence, limit functional or structural deterioration, prevent later complications, relieve current distress, deliver reassurance, or allow to die with comfort and dignity [52]. However, this list of goals relies on weakly defined categories. JP. Boissel and P. Gallois have suggested hierarchizing therapeutic goals according to their usefulness. They have thus defined the following five levels: increasing life expectancy, decreasing the occurrence of non-lethal morbid events, getting rid of inconvenient symptoms, preventing handicaps, and improving quality of life [20]. This classification does not include the curing and remission goal. Other authors have developed a goal typology with four categories: three were professional goal categories (functional, biological and adaptive) and the other category was defined by the patient. To limit the tensions between professionals’ and patients’ personal goals, they proposed to place the personal goals at the top of the goal hierarchy and then to translate them into professional goals [53]. To our knowledge, none of these classifications have been tested in care situations.

Several qualitative studies based on interviews of various actors involved in the medical process have been conducted, with the aim of describing care goals. A study of elderly patients with multimorbidities showed that the goals expressed by the patients, their family physicians and informal caregivers were grouped according to the following categories: health maintenance, health improvement and symptoms management, behavior change, preparation for future needs, social help, safety and dignity [21]. A study in a geriatric evaluation center on family caregivers and clinicians showed that the most important goals were as follows: day-to-day functioning, safety, emotional and behavioral issues, caregiver stress, medical issues, and social support [22]. In a study in a geriatric ambulatory consultation center, the patients reported six categories of goals: safety, independence and day-to-day functioning, social and family relationships, personal health, economic stability, and maintenance of autonomy and dignity [23]. In another study targeting patients with dementia, the same team identified two additional categories: general well-being and behavioral and emotional issues [24]. The results of these qualitative studies shed light on the patients’ perspectives, but the reported categories have been built empirically and cannot be considered as operational classifications.

Our proposal to use an eight-category generic classification proved operational in daily general practice, although it remains to be further validated. It may be extended with terminologies that include detailed goals, such as the International Classification of Primary Care (ICPC-2) to illustrate the morbidity risk reduction categories [27] or the International Classification of Functioning (ICF) to illustrate the improvement or maintenance of functioning [54].

### Strengths and limitations

There were no missing data in our database. Classification mistakes were limited by the training of the investigators, data control and the use of an online coding software. The mistake rate for data entry was estimated at around 3.0% for drugs and for goals.

The study was conducted with university trainers, but the characteristics of their patients and drug prescriptions can be considered broadly representative of patients attending general practice [55]. The study is only about the goals of drug prescriptions and does not encompass all other care goals. It would be interesting for future studies to focus on prescription goals for care delivered by allied health care professionals (e.g., nursing care, physiotherapy, podiatry, ergotherapy, or speech therapy), medical devices, sick leave periods and home support services.

The study period over fall and winter months could increase the frequency of viral infections recorded. As they are usually treated symptomatically, this could eventually overestimate the frequency of symptomatic goals.

Finally, the purely observational design of this exploratory study prevents causal inference. The restriction to the main goal for each of the drugs prescribed might have underestimated the agreement rate between the patients and the physicians as well as the Kappa coefficient.

## Conclusions

This study shows that the physicians’ main goal of prescribing drugs is mostly to relieve symptoms and that it is poorly shared with patients. Making the prescription goals more explicit by the physician is a prerequisite to sharing the treatment decision with the patient. It remains to be assessed, however, whether it is possible to collect and discuss information on prescription goals on a daily basis. The increasing use of patient-centered outcome criteria in drug trials will support collaborative prescription goal setting.

## Supplementary information

**Additional file 1.** Questionnaire. questionnaire used in the study.

## Data Availability

The dataset analyzed during the current study is available from the corresponding author on reasonable request.

## Abbreviations

ATC: Anatomical Therapeutic Chemical classification of drugs
ICF: International Classification of Functioning disability and health
ICPC: International Classification of Primary Care
