# Supplementary material for: Drug prescription goals in primary care: a cross-sectional study
Source: BMC Health Serv Res. 2020 Jan 2;20:6. doi: 10.1186/s12913-019-4870-y (PMC6941394; doi:10.1186/s12913-019-4870-y)
Supplement: Supplementary file 1 — Additional file 1. Questionnaire. questionnaire used in the study. [file 12913_2019_4870_MOESM1_ESM.pdf]

Start time	End time	GP's initials	Date of encounter	Encounter	Patient	Birth year	Gender	Student
/__ __:__ __/	/__ __ :__ __/	/__ /__/	/__ __/__ __/__ __/	Office ☐ Home ☐	New ☐ Known ☐	/__ __ __ __/	M ☐ F ☐	Yes ☐ No ☐

Medical fee exemption status	Occupation
For long-term condition ☐<br>For low income ☐	Farmer ☐ craftsman, trader, manager ☐ employee ☐ worker ☐<br>senior executive, intellectual profession ☐ intermediate profession ☐ retired person ☐ other without professional activity ☐<br>.

Health problem assessment (symptom of diagnostic) [**HPA**]

1.	5.
2.	6.
3.	7.
4.	8.

Drug prescription (Initiation ou renewal)

INN or brand name	I / R	N°HPA	Route	Main objective	INN or brand name	I / R	N° HPA	Route	Main objective
1.					11.				
2.					12.				
3.					13.				
4.					14.				
5.					15.				
6.					16.				
7.					17.				
8.					18.				
9.					19.				
10.					20.				

I= Initiation; R= Renewal

Main objective: 1= to decrease the risk of mortality (all-cause or cause-specific); 2= to decrease the risk of morbidity (disease or complications); 3= to cure or provide remission of disease; 4= to relieve symptoms; 5= to improve or maintain quality of life; 6= to improve or maintain functioning; 7= other goal to specity (in free text) ; 8= no goal
